# Cryptophyta as major bacterivores in freshwater summer plankton

**DOI:** 10.1038/s41396-018-0057-5

**Published:** 2018-02-20

**Authors:** Vesna Grujcic, Julia K Nuy, Michaela M Salcher, Tanja Shabarova, Vojtech Kasalicky, Jens Boenigk, Manfred Jensen, Karel Simek

**Affiliations:** 1 Biology Centre of the Czech Academy of Sciences, Institute of Hydrobiology, Na Sádkách 7, 37005 Ceske Budejovice, Czech Republic; 2 Faculty of Science, University of South Bohemia, 37005 České Budějovice, Czech Republic; 3 Biodiversity, University Duisburg-Essen, 45117 Essen, Germany; 4 Limnological Station, University of Zurich, 8802 Kilchberg, Switzerland

## Abstract

Small bacterivorous eukaryotes play a cardinal role in aquatic food webs and their taxonomic classification is currently a hot topic in aquatic microbial ecology. Despite increasing interest in their diversity, core questions regarding predator–prey specificity remain largely unanswered, e.g., which heterotrophic nanoflagellates (HNFs) are the main bacterivores in freshwaters and which prokaryotes support the growth of small HNFs. To answer these questions, we fed natural communities of HNFs from Římov reservoir (Czech Republic) with five different bacterial strains of the ubiquitous betaproteobacterial genera *Polynucleobacter* and *Limnohabitans*. We combined amplicon sequencing and catalyzed reporter deposition fluorescence in situ hybridization (CARD-FISH) targeting eukaryotic 18 S rRNA genes to track specific responses of the natural HNF community to prey amendments. While amplicon sequencing provided valuable qualitative data and a basis for designing specific probes, the number of reads was insufficient to accurately quantify certain eukaryotic groups. We also applied a double-hybridization technique that allows simultaneous phylogenetic identification of both predator and prey. Our results show that community composition of HNFs is strongly dependent upon prey type. Surprisingly, Cryptophyta were the most abundant bacterivores, although this phylum has been so far assumed to be mainly autotrophic. Moreover, the growth of a small lineage of Cryptophyta (CRY1 clade) was strongly stimulated by one *Limnohabitans* strain in our experiment. Thus, our study is the first report that colorless Cryptophyta are major bacterivores in summer plankton samples and can play a key role in the carbon transfer from prokaryotes to higher trophic levels.

## Introduction

Heterotrophic nanoflagellates (HNFs) undoubtedly belong to the most abundant eukaryotes on Earth, inhabiting freshwaters, oceans, sediments, and soils [1–4]. They are particularly abundant in planktonic communities, acting as primary prokaryotic grazers and thus playing an essential role in nutrient cycling [5–9]. They also represent the most important link between dissolved organic matter and its transfer through growing bacterial cells to higher trophic levels [10–12]. Despite their importance and abundance they have received less attention than prokaryotes [13, 14] and their diversity has been generally less investigated in freshwaters [15, 16] than in oceans [1, 3, 17, 18]. Furthermore, knowledge of which species or taxa are the most important bacterivores in freshwaters and which bacteria are actually consumed by these small protists still remains poorly understood [12, 19, 20]. Some studies however, pointed to the importance of flagellates related to *Spumella* spp., that rapidly respond to sudden bacterial prey amendments ([20]; see also [21–23]), implying that these flagellates are significant bacterivores.

Furthermore, small size and inconspicuous morphology of HNFs makes them hard to be identified via classical epifluorescence microscopy but the advance of high-throughput sequencing (HTS) facilitated an easier taxonomic classification of these smallest eukaryotes [14, 15, 17, 24]. While HTS represents an efficient tool for an identification of different taxa in a sample, one of the main problems of this approach is how well the number of reads obtained by HTS corresponds to the real cell abundance [25–27]. A method enabling microscopic visualization and thus providing a more accurate quantification of specific cells, by using oligonucleotide probes as phylogenetic markers, is catalyzed reporter deposition fluorescence in situ hybridization (CARD-FISH). Altough there are many publications exploiting HTS [15, 28–30] or CARD-FISH approaches [31–37] to analyze microbial eukaryotic communities, a combination of both methods has rarely been used [25].

Contrasting to flagellates, abundance and diversity of bacteria in freshwaters is well documented, indicating the dominance of a few ubiquitous phylogenetic lineages of Alphaproteobacteria and Betaproteobacteria, Actinobacteria and Bacteroidetes [38]. Among Betaproteobacteria, the genera *Limnohabitans* [39] and *Polynucleobacter* [40] are very abundant members of freshwater plankton(i.e., those to be most likely met in planktonic environments by flagellates). Previous research showed that some bacteria of the genus *Limnohabitans* induced prey-specific differences in flagellate growth parameters [41], which influenced the community composition of flagellates [20] Although *Limnohabitans* and *Polynucleobacter* are both highly abundant in a broad array of habitats, they exhibit contrasting lifestyles [42]. *Limnohabitans* have high growth rates and limited morphological versatility in situ [43, 44] which makes them highly vulnerable to protistan grazing [19, 43, 45]. They possess generally larger genomes (2.5–4.9 Mb [46, 47], a high metabolic flexibility [39, 48], and larger mean cell volumes compared to other planktonic prokaryotes [39, 43, 49]. In contrast to *Limnohabitans*, members of the *Polynucleobacter* genus have medium-sized genomes (2.0–2.4 Mbp; [50, 51]), a generally smaller cell size, and a more passive lifestyle relying on photodegradation products of humic substances [52]. However, data on in situ grazing-induced population turnover rates of these bacteria is still missing [52]. All the above mentioned characteristics of the two bacterial groups makes them suitable models for testing carbon flow to higher trophic levels.

We can assume that certain bacterial taxa, especially those with high growth and grazing-induced mortality rates, should have a prominent role in carbon flow (acting as “link” [53]) to higher trophic levels in a particular environment. Thus, the growth parameters of natural HNF communities feeding on such taxa can be used as a measure of carbon flow from a specific bacterial group to grazers and, furthermore, of the food quality of a particular bacterial prey for HNF. It has already been demonstrated that not all bacteria stimulate the growth of HNF in the same way and their growth efficiencies directly affect the carbon flow to higher trophic levels [20]. We thus assume that prey quality and availability can severely influence the community composition of HNF.

In this study, we conducted short-term manipulation experiments by the addition of different strains of planktonic Betaproteobacteria to a natural HNF population. Since bacterivorous flagellates and bacteria grow with approximately the same high growth rates in plankton environments (~10 h doubling time (DT), [54]) short-term experiments with high sampling frequency allowed us to efficiently track major trends in growth and community responses of HNF amended by different prey. We combined amplicon sequencing of 18 S rRNA genes and CARD-FISH with newly designed probes based on amplicons to quantify and visualize major freshwater flagellate bacterivores. We also applied a double-hybridization technique, developed by ref. [55] and advanced in this study to verify taxonomic affiliations of both grazers and prey at the same microscopic preparation. This approach is, to our knowledge, rarely used in current microbial ecology. With these techniques we intended to address the following aims: (a) to investigate the effects of different bacterial prey characteristics on the growth of natural freshwater bacterivorous flagellates, (b) to examine which flagellate taxa are key bacterivores in experimental treatments, based both on abundances and specific grazing rates of prominent HNF lineages, (c) and finally to examine the quantitative match between HTS and CARD-FISH targeting prominent flagellate bacterivores in our prey-amended treatments.

## Materials and methods

### Experimental design

We applied a similar experimental design to that detailed in refs. [20] and [41]. Plankton samples were collected from 0.5 m depth from the mesoeutrophic Římov reservoir, South Bohemia, Czech Republic (48°50′46.90″N, 14°29′15.50″E, for more details see [56]) at the late summer phytoplankton bloom on 18 August 2014 (water temperature 20.3 °C). Water was gravity filtered through 5 µm pore-size filters to release the flagellate community from grazing pressure of zooplankton and larger predatory flagellates and ciliates. The 5-µm treatment represented a simplified prokaryote-HNF food chain supposedly dominated by small, primarily bacterivorous nanoflagellates [44]. Samples were preincubated at 18 °C for 12 h, which resulted in approximately twofold increases in HNF abundance, and slight decreases in the number of free-living bacteria (~1 × 10^6^ ml^−1^). Our experimental set-up was composed of five different treatments, each of them separately amended with distinct bacterial prey: two with strains of *Polynucleobacter* lineage PnecC (PnC6 and PnC1, for details see Table 1) and three with strains belonging to different lineages of *Limnohabitans* spp. (T6-5, Rim47, and Rim11; Table 1) [39]. These bacteria differed markedly in cell shape and size (Table 1). All five bacterial strains were pre-grown in nutrient- (i.e., CNP) rich liquid medium (3 g l^−1^ NSY) [57], pelleted by centrifugation, washed and resuspended in 0.2 µm filtered, and sterilized water from Římov reservoir as detailed in refs. [20] and [41].

**Table 1:** Morphological characteristics of bacterial strains used as a prey for natural HNF communities in the experiments

Species	Strain	Lineage	Volume (µm^3^)	Length (µm)	Cell shape
*Polynucleobacter* sp.	PnC1 (czRimovFAM-C1)	PnecC	0.057	0.88	Small solenoid
*Polynucleobacter* sp.	PnC6 (czRimov8-C6)	PnecC	0.049	0.58	Short rod
*Limnohabitans* sp.	Rim11	LimB	0.051	0.63	Short rod
*Limnohabitans* sp.	Rim47	LimC4	0.055	0.66	Coccoid
*Limnohabitans* sp.	T6-5	LimC	0.472	2.21	Thin curved rod

Treatments were separately amended with solutions of prey bacteria added at ~10 times higher concentrations compared to natural background bacterial abundances. Since the prey bacteria differed in cell sizes (Table 1), the additions of the strains were set to yield approximately the same initial biovolumes for all strains [20, 41]. The experiments were run in triplicates and treatments were kept at 18 °C in the dark, since the target bacterivorous grazers were purely HNFs. The treatments containing only natural bacteria and protists present in the original samples served as controls, compared to the prey enriched treatments (referred to as PnC1, PnC6, T6-5, Rim-47, and Rim-11 throughout the text; Table 1). Subsamples for detection of HNF, bacterial abundances, and biovolumes were aseptically taken in a laminar flow hood at 12–24 h intervals. Additional samples were taken at selected time points for fluorescence in situ hybridization followed by catalyzed reporter deposition (*t*_0_, *t*_40_, and *t*_66_), and for collecting DNA for sequencing (*t*_0_ and *t*_40_).

### Enumeration and biovolume estimation of bacteria and HNFs

Samples (15–20 ml) fixed with formaldehyde (2% final concentration) were used for the enumeration of bacteria (0.5–2 ml subsamples) and HNF (4–10 ml subsamples) on 0.2-µm and 1-µm pore-sized filters (Osmonics, Inc., Livermore, CA), respectively. All samples were stained with DAPI (4′, 6-diamidino-2-phenylindole, at a final concentration of 1 µg ml^−1^) and microbes were counted via epifluorescence microscopy (Olympus BX 60). Bacterial biovolumes were measured by using a semiautomatic image analysis system (NIS-Elements 3.0, Laboratory Imaging, Prague, Czech Republic). To calculate mean cell volumes of HNF (approximated to prolate spheroids [41]), lengths and widths of the 50 cells in each triplicate treatment were measured manually on-screen with a built-in tool of the image analysis system (NIS-Elements 3.0).

A treatment-specific HNF cell number increase was used to calculate maximum HNF growth rate, DT, length of lag phase, and relative growth rate as detailed in ref. [41]. In brief, maximum HNF growth rate was calculated based upon the equation for exponential growth, lag phase was calculated as the period from *t*_0_ to the intercept between the best fit line of HNF growth and the zero-time level of HNF abundance. Volumetric gross growth efficiency (GGE) was based on comparisons of HNF versus bacterial biovolumes (for details see refs. [41, 54]). Relative growth rates were derived from relating the HNF time course data from all treatments to the treatment where the most rapid growth of HNF was recorded.

### Illumina sequencing of eukaryotic communities and data analysis

Genomic DNA was extracted from biomass collected on 0.2 µm-pore-size filters (47 mm diameter; Osmonics) employing a phenol–chloroform extraction and subsequent ethanol precipitation. DNA was extracted from triplicates collected at *t*_0_ and *t*_40_ hours of experiment. PCR amplification was conducted with indexed primers targeting an amplicon of 450 bp in the hypervariable V9 region of the SSU and the ITS1 region of the eukaryotic rRNA gene. Forward and reverse primers used are Euk1391F 5′-GTA CAC ACC GCC CGT C-3′ [58] and ITS2 5′-GCT GCG TTC TTC ATC GAT-3′ [59]. Amplification was performed with a BioRad T 100 cycler with a 25 µl mix containing 2 U Phusion High Fidelity Polymerase (Finnzymes, Oy, Espoo, Finland), 5 µl of 5 × HF buffer, 0.25 pM of each primer, 200 µM of each desoxyribonucleosidtriphosphate, 0.5 µl DNA template and 17.25 µl water. Concentration of DNA template ranged between 12 and 60 ng µl^−1^. The amplification protocol was performed with 30 s initial denaturation at 98 °C followed by 35 PCR cycles comprising 98 °C for 10 s, 57° C for 20 s, 72° C for 35 s, and a single final elongation step for 10 min at 72° C. The amplification of each sample was performed in five replicates to increase the total concentration per sample. The pooled and indexed samples were pair-end-sequenced by Eurofins (Eurofins Genomics, Germany, Ebersberg) with an Illumina MiSeq instrument using V3 chemistry.

Raw sequence reads were demultiplexed, quality filtered, clustered, and assigned to taxonomy according to ref. [58] with the following modifications: low quality tails were removed, reads with an average Phred quality score <25 were trimmed [60]. As the 3′ ends were of overall low quality, we decided to trim the reads to 89 nucleotides, and all reads with at least one base with a Phred quality score of <15 were removed. As the reverse reads had significantly lower quality than the forward reads, we decided to analyze only the single-end reads to avoid quality based biases of reverse reads in the community analysis. The single-end reads were quality filtered using PANDASeq version 2.7. Reads with uncalled bases were discarded. Chimeras were identified and discarded using UCHIME. The remaining sequences were clustered to OTUs with SWARM (swarm v2.1.6 [61]) and assigned to taxonomic information using BLAST 2.2.30+ [62] requiring 85% identity and an evalue cutoff of 1e−^12^. Heterotrophic flagellates were selected by definitions from ref. [63] including only groups which are known to be mostly heterotrophic and to possess flagella. The amplicon data used in this study are accessible in the sequence read archive of the NCBI database as BioProject PRJNA385800.

### Phylogenetic tree reconstruction and design of novel oligonucleotide probes

Representative amplicons of the 30 most abundant OTUs were aligned with the SINA aligner [64] and imported into ARB [65] using the SILVA database SSURef_NR99_123 [66]. Alignments were manually refined and a maximum likelihood tree (1000 bootstraps) including their closest relatives was constructed on a dedicated web server [67]. Oligonucleotide probes targeting all Katablepharidophyta and the CRY1 lineage of Cryptophyta [35] were designed in ARB using the tools probe_design and probe_check and evaluated with the web tool mathFISH [68]. A similar probe for the CRY1 lineage was also designed by ref. [35], targeting exactly the same 18 S rDNA sequences and being equal in terms of coverage and specificity.

### Catalyzed reporter deposition fluorescence in situ hybridization

The CARD-FISH protocol [69] was used with specific oligonucleotide probes targeting all Betaproteobacteria (BET42a [70]), all *Limnohabitans* strains used in this study (R-BT065 [39, 44]) and *Polynucleobacter* lineage PnecC (PnecC-16S-445, [71]), respectively. Fluorescein-labeled tyramides (Invitrogen Corporation, Carlsbad, CA) were used for signal amplification and an epifluorescence microscope (Olympus BX 60 microscope) for visualization. We checked for ingestion of prey bacteria in HNF food vacuoles [19] in all experimental treatments at times *t*_40_ and *t*_66_ h.

Moreover, CARD-FISH was applied for HNF following a previously published protocol [72]. We used the general probes Euk516 targeting all eukaryotes [73, 74], CryptB targeting Cryptophyta [75], and two newly designed probes specific for the CRY1 lineage of Cryptophyta (CRY1-652) and Katablepharidophyta (Kat-1452, for details see Table 2). Probe CryptB covers >80% of all Cryptophyta, including the CRY1 lineage, but does not target Katablepharydophyta. Probe Euk516 [73] targeted an average of 89.6% DAPI-stained eukaryotes. The newly designed probes were tested with different formamide concentrations in the hybridization buffer until highest stringency was achieved at 30% and 60% for probes CRY1-652 and Kat-1452, respectively. After signal amplification with fluorescein-labeled tyramides (Invitrogen Corporation, Carlsbad, CA), filters were counterstained with DAPI and evaluated in an epifluorescence microscope (Olympus BX 60).

**Table 2:** Details of CARD-FISH probes used in this study. See Figure S3 for details in the phylogenetic positioning of probes Cry1-652 and Kat-1452

Probe name	Target	Sequence (5′–3′)	% Formamide	Reference
Euk516	All eukaryotes	ACCAGACTTGCCCTCC	20%	[73]
CryptB	Cryptophyta	ACGGCCCCAACTGTCCCT	50%	[75]
Cry1-652	CRY1 lineage	TTTCACAGTWAACGATCCGCGC	30%	This study
Kat-1452	Katablepharidophyta	TTCCCGCARMATCGACGGCG	60%	This study

Bacterial probes R-BT065 [44] and PnecC-16S-445 (71), and eukaryotic probes CryptB [75], CRY1-652, and Kat-1452 (Table 1) were also used for a double hybridization of prey and grazers in parallel [55] followed by amplification with fluorescein (probe R-BT065) and Alexa546 (probes CryptB, CRY1-652 and Kat-1452) labeled tyramides (Invitrogen Corporation, Carlsbad, CA), respectively.

### Bacterivory rates of heterotrophic nanoflagellates at *T*_0_

Grazing rates of the HNF community present in the unfiltered reservoir sample used for the experiment (*T*_0_) were examined by using fluorescently labeled bacteria (FLB [53]) prepared from a mixture of *Limnohabitans* sp. from the LimC lineage [39] and two strains from the PnecC lineage of *Polynucleobacter* isolated from the reservoir. HNF bacterivory was determined in short-term FLB direct-uptake experiments in combination with fluorescence microscopy as detailed in ref. [44]. To estimate total HNF grazing, we multiplied the average uptake rates of HNF by their in situ abundance at *T*_0_.

In addition, we quantified the average number of DAPI-stained bacteria, as well as bacteria targeted by the general probe EUB I-III [76], in food vacuoles of bacterivorous HNF targeted by different CARD-FISH probes in the unfiltered samples from *T*_0_. We applied the general probe for Eukaryotes (Euk516) and compared it to the food vacuole contents of HNF targeted by probes for Cryptophyta and its CRY1 lineage. The combination of these methods allowed estimating cell-specific (expressed as number of bacteria ingested per flagellate cell) and bulk bacterivory rates of total HNF compared to different flagellate lineages. The proportion of bacterial-standing stock removed per day was used as a proxy of the significance of the total grazing impact of the different flagellate groups in untreated reservoir water (see Table 3 for details).

**Table 3:** Grazing characteristics of different flagellate groups at time *T*_0_ from Rimov reservoir

	HNF 10^3^ ml^−1^	HNF (%)	Bact flag^−1^	IGR at *T*_0_ bac HNF^−1^ (h)	TGR per day 10^6^ml^−1^ (d)	Bact standing stock grazed per day (%)	% of total TGR of HNF
All HNF	5,4	100	2,9	13,7	1,78	54,2	100
All Crypto	3,38	63	3,1	14,8	1,2	36,6	70
CRY1 lineage	0,1	1,8	1,8	8,5	0,02	0,6	1,1

*IGR* individual cell-specific grazing rate; *TGR* total grazing rate calculated for the whole HNF community and of its FISH probe defined subgroups (Crypto and CRY1 lineage). Bact flag^−1^ represents average number of bacteria stained with general EUB I-III probe per group of flagellate [76]

### Statistical analysis

Statistical analyses were performed with the Excel stats package (Microsoft Office Professional Plus 2010, Santa Rosa, CA, USA, Version 14.0.7128.5000). We analyzed the effects of strain characteristics on HNF GGE, DT, lag phases, and relative growth rates by two way analysis of variance (ANOVA) followed by post hoc Tukey tests. The same analysis was applied for comparing differences in percentage of hybridized flagellate cells between time *t*_0_, *t*_40_, and *t*_66_ and differences in percentages of flagellate reads between treatments. *t* tests were used for identifying differences between percentages of hybridized cells with CryptB and Kat-1452 probes and percentages of reads belonging to the same groups.

## Results

### Time course changes in bacteria and HNF

**Fig. 1:**
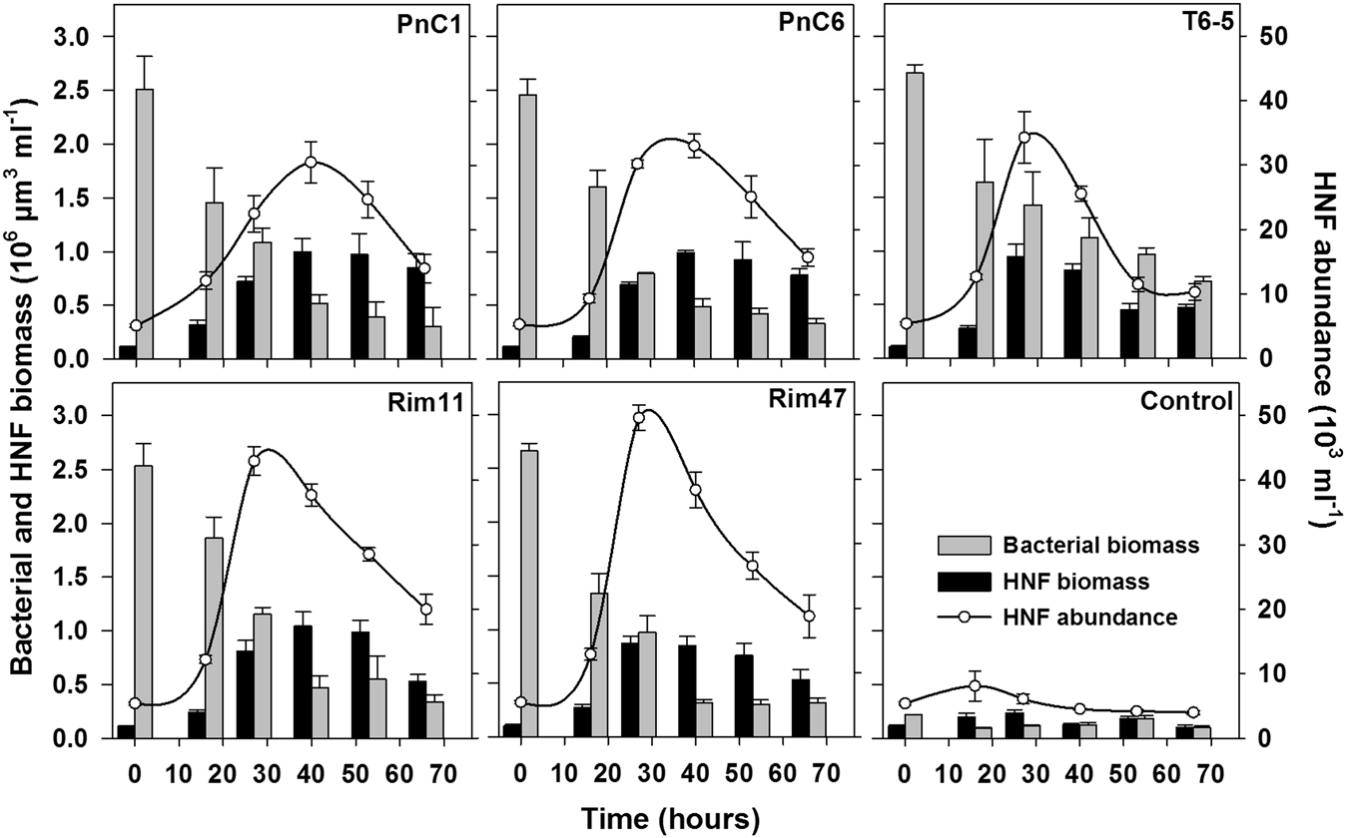
Time course changes in HNF abundances, HNF, and bacterial biovolumes in all treatments. Values are means of triplicates; error bars show SD

**Fig. 2:**
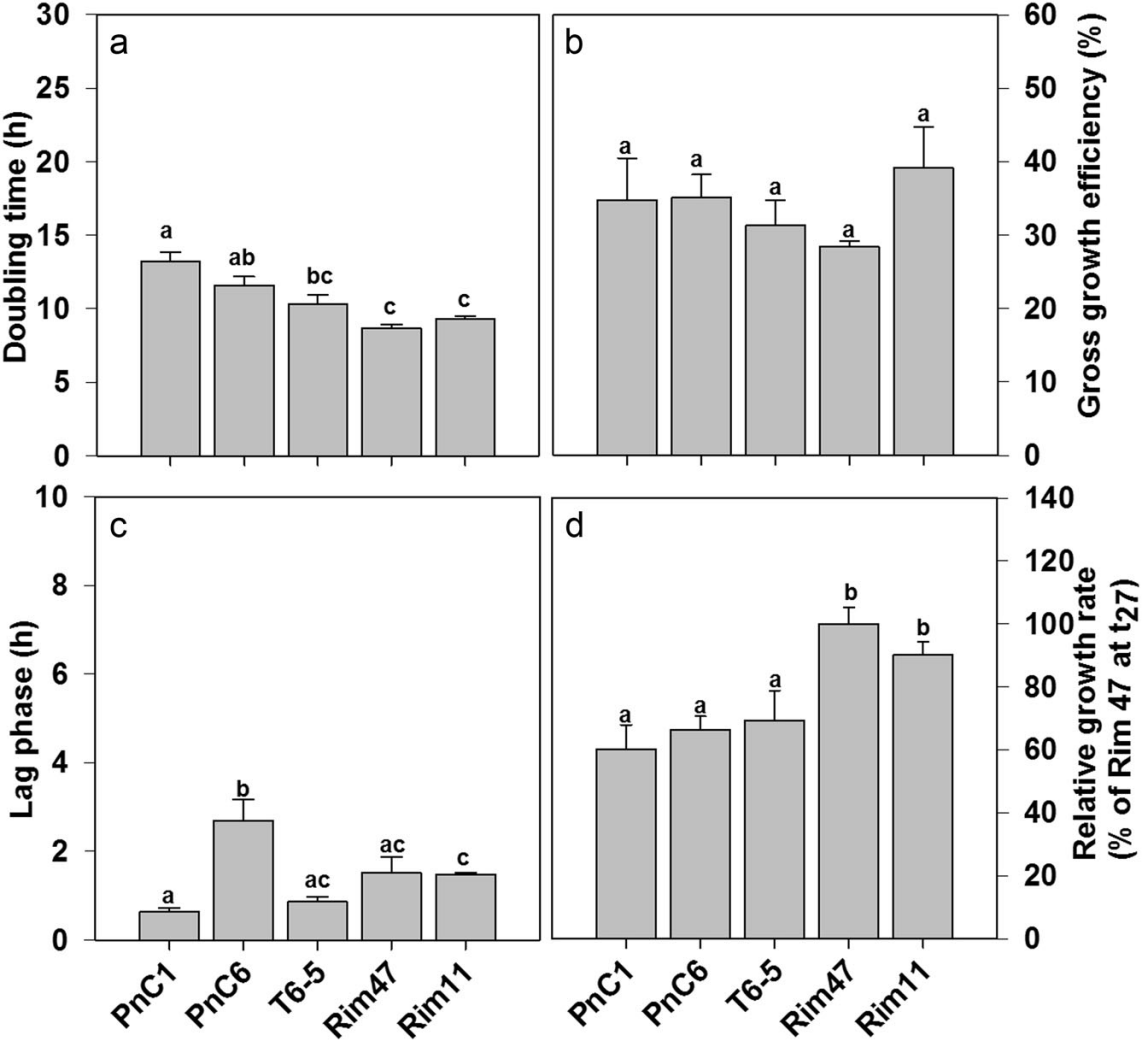
Doubling times **a**, gross growth efficiencies **b**, lag phases **c**, and relative growth rates **d** of HNFs in all treatments amended with bacterial strains. Values are means of triplicates, error bars show SD. Different letters above bars denote significant differences (two way ANOVA followed by post hoc Tukey test)

We tested growth responses of natural HNF communities to amendments with five different bacterial strains. While the strains differed in size, morphology, and taxonomic affiliation (Table 1), they all were swiftly consumed by the grazer HNF community (Supplementary Figure 1) and thus also supported significantly more rapid growth of natural HNF communities compared to the control (Figs. 1, 2). Numbers and biomasses of bacteria and HNF remained relatively stable in control treatments, except for a slight increase of HNF within the first 16 h (Fig. 1; Supplementary Figure 1). Temporal changes in biovolumes of HNF and bacteria roughly corresponded to the treatment-specific trends observed for abundances (Fig. 1).

Bacterial numbers and biovolumes started to decrease markedly after 16 h in most of the prey amendments, except for treatments T6-5 and Rim47, where bacteria decreased already shortly after the beginning of the experiment (Supplementary Figure 1). Notably, bacterial numbers and biomasses slightly increased before the onset of HNF growth in two treatments (PnC6 and Rim11, 0–16 h; Fig. 1, Supplementary Figure 1). HNF abundances increased from the initial ~5 × 10^3^ to ~30–50 × 10^3^ cells ml^−1^ in treatment-specific fashions (Fig. 1). Generally, maxima were achieved at *t*_27_ h, except for treatments PnC1 and PnC6 (the *Polynucleobacter* strains) where peaks occurred later (*t*_40_ h), followed by a subsequent decrease (Fig. 1).

### HNF growth parameters

Similar initial biovolumes of the distinct bacterial strains yielded different HNF growth dynamics. The fastest growing HNFs were those feeding on *Limnohabitans* strains Rim47 (DT = 8.6 h) and Rim11 (DT = 9.3 h). DTs of these flagellates were significantly different (*p* < 0.001, one way ANOVA followed by Tukey test) from DTs of HNFs growing in treatments PnC1 (DT = 13 h) and PnC6 (DT = 11.5 h). HNF growth in treatment T6-5 (DT = 10.3 h) was significantly different only from that in treatment PnC1 (Fig. 2a). Lag phases of HNFs were relatively short (0.6 – 3 h) with treatment PnC6 having significantly longer DT than all other treatments (*p* < 0.005). HNFs in treatment PnC1 had significantly shorter lag phase than HNFs in treatment Rim11. However, there was no significant difference in length of lag phase between treatments containing the three *Limnohabitans* strains (T6-5, Rim47, and Rim11; Fig. 2c). Volumetric GGEs of flagellates ranged from 28 to 39% (Rim47 and Rim11, respectively) and showed no significant difference between different prey items (Fig. 2b).

Relative growth rates related to the increase of HNF numbers in treatment Rim47 (the most rapid cell number increase at *t*_27_ h, set as 100%) were significantly lower in treatments PnC1, PnC6, and T6-5 than in Rim11 and Rim47, suggesting that the latter two strains represented the best food supporting rapid HNFs growth in combination with the shortest DTs (Fig. 2d).

### Effects of bacterial prey on the composition of HNF

**Fig. 3:**
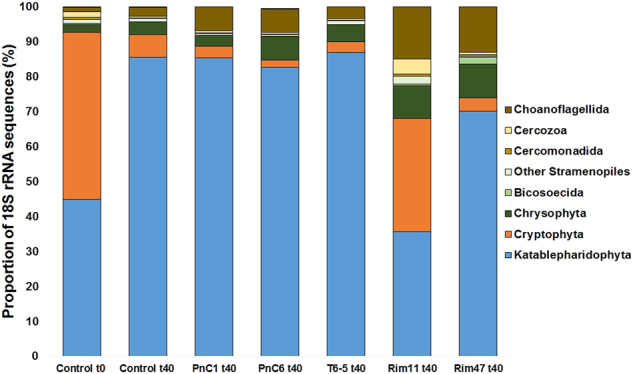
Percentage of reads belonging to different taxonomic groups of protists in all treatments at different time points. Control *t*_0_ represents the starting community from the reservoir. Control *t*_40_ represents the control after 40 h of experiment, PnC1 *t*_40_, PnC6 *t*_40_, T6-5 *t*_40_, Rim47 *t*_40_ and Rim11 *t*_40_ are treatments amended with different bacterial strains after 40 h of experiment. Values expressed as percentages are means of triplicates

The 18 S *rRNA* gene amplicon data set comprised 3,527,902 reads that were filtered for bacterivorous flagellate groups. A total of 1,576,480 reads related to flagellates were analyzed, with the most abundant group belonging to Katablepharidophyta, accounting for 35–85% in the different treatments (Fig. 3).

We compared relative proportions of reads assigned to heterotrophic flagellate groups at *t*_0_ h (control *t*_0_) of the experiment to treatments after 40 h of the experiment (Fig. 3). The initial sample from Římov reservoir was composed of 47% Cryptophyta, 44% Katablepharidophyta, and low percentages (<2%) of Chrysophyceae, Bicosoecida, other Stramenopiles, Cercomonadida, Cercozoa, Choanoflagellida, and Haptophyta. After 40 h of experiment (control *t*_40_), the initial sample changed significantly (*p* < 0.001), with Cryptophyta decreasing to 6% and Katablepharidophyta increasing to 84%. Chrysophyta accounted for 4% and Choanoflagellida for 2% of the flagellate reads while other groups stayed more or less stable or almost disappeared (Fig. 3).

In the treatment amended with strain PnC1, Katablepharidophyta dominated the analyzed sample with 85% of reads, while only 3% belonged to Cryptophyta, 7% to Choanoflagellida and 3% to Chrysophyta. Relatively, similar shifts in major flagellate groups occurred also in treatments PnC6 and T6-5, while other groups such as other Stramenopiles and Cercozoa accounted for <1% (Fig. 3). Treatments Rim11 and Rim47 displayed more marked changes with a significant increase (*p* > 0.001) in the proportions of flagellates representing typical bacterivorous groups such as Chrysophyta and Choanoflagellida. Treatment Rim47 had 68% of reads belonging to Katablepharidophyta, 4% to Cryptophyta, 13% to Choanoflagellida, 9% to Chrysophyta, and ≤2% to Bicosoecida, Cercomonadida, and Cercozoa. In contrast, treatment Rim11 was most distinct (Fig. 3), with 35% of reads belonging to Katablepharidophyta, 32% to Cryptophyta, 15% to Choanoflagellida, 9% to Chrysophyta, 4% to Cercozoa, and ≤2% to other Stramenopiles, Bicosoecida, and Cercomonadida.

**Fig. 4:**
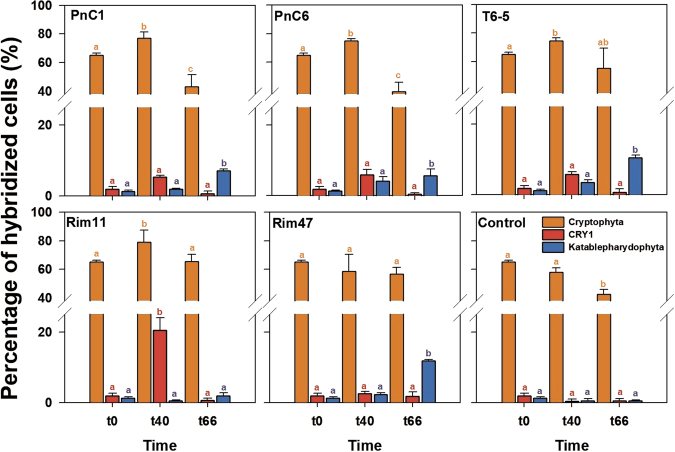
Relative abundances of cells hybridized with probes targeting all Cryptophyta, lineage CRY1, and all Katablepharidophyta at three different time points: *t*_0_, beginning of experiment, representing the starting community from the reservoir; *t*_40_ and *t*_60_ represent proportions after 40 and 60 h of experiment. Different letters above the columns indicate significant differences between different times of the experiment within one treatment targeted with one probe (post hoc Tukey test). Values are means of triplicates, error bars show SD

**Fig. 5:**
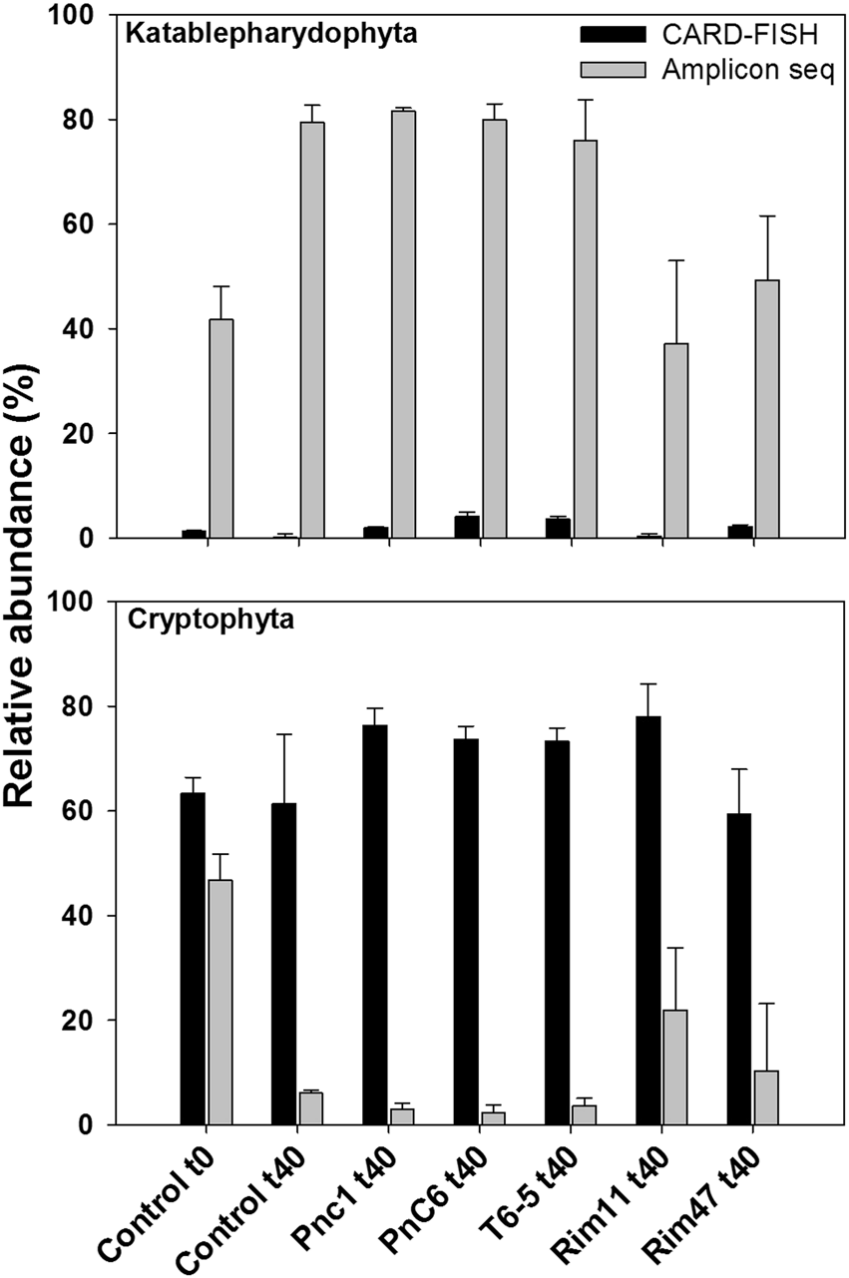
Comparison of relative abundances of 18 S rDNA amplicon reads and relative abundance of cells get by CARD-FISH. Differences were significant for all treatments (*t* test *p* < 0.001). Values are means of triplicates, error bars show SD

**Fig. 6:**
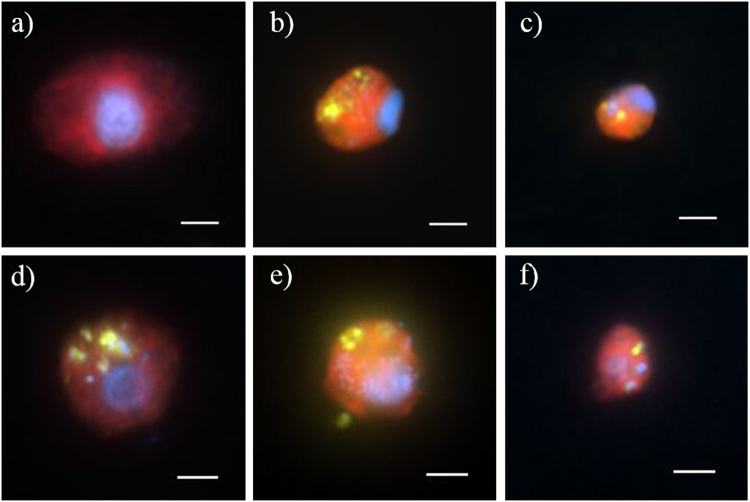
Double hybridization of bacterial prey and HNF predator. Each image is an overlay of three pictures of the same HNF cell observed under ultraviolet excitation (showing the blue nucleus after DAPI staining), green light excitation (red color corresponding to different HNF groups labeled with Alexa546 using CARD-FISH) and blue light excitation (yellow-green color corresponding to fluorescein-labeled *Limnohabitans* spp. or *Polynucleobacter* cells in food vacuoles after CARD-FISH with probe R-BT065 or PnecC-16S-445, respectively). Scale bar is 2 µm. **a** HNF hybridized with probe Kat-1452 targeting all Katablepharydophyta, **b** bacteria and HNF hybridized with probes R-BT065 targeting *Limonhabitans* and CRY1-652 targeting the CRY1 lineage of Cryptophyta, **c** bacteria and HNF hybridized with probes PnecC-16S-445 targeting *Polynucleobacter* and CRY1-652 targeting the CRY1 lineage of Cryptophyta to (**d** and **e**) bacteria and HNF hybridized with probes R-BT065 targeting *Limonhabitans* and CryptB targeting all Cryptophyta **f** bacteria and HNF hybridized with probes PnecC-16S-445 targeting *Polynucleobacter* and CryptB targeting all Cryptophyta

Percentages of flagellates targeted by specific CARD-FISH probes revealed highly significant differences compared to the proportions derived from amplicon sequencing (Figs. 4, 5; Supplementary Figure 3). Relative abundances of flagellates belonging to Katablepharidophyta were 1.5% at time *t*_0_. These flagellates increased significantly (*p* < 0.001; from 6.3 to 11.8%) until the end of the experiments (*t*_66_) in most treatments, except for the Rim11 and control, where they represented relatively stable proportions (Fig. 4). Flagellates affiliated to Cryptophyta accounted for 62.5% of all HNFs at time *t*_0_ (Table 2). After 40 h, their proportion increased significantly (*p* < 0.001) to >70% in treatments PnC1, PnC6, T6-5, and Rim11, while they slightly decreased in treatment Rim47 and in the control. At *t*_66_ h, the proportions significantly decreased in PnC1 and PnC6 treatments, while in other treatments their proportions remained stable or slightly decreased compared to *t*_0_. Relative abundances of flagellates belonging to the CRY1 clade of Cryptophyta were 1.8% at *t*_0_ and after 40 h this proportion significantly increased to 20.5% in Rim11 (*p* < 0.001) and also slightly rose in all other treatments. At the end of the experiments, proportions of CRY1 significantly decreased in all treatments to 0.3–1.8% (Fig. 4). The pronounced growth of Cryptophyta was also visible in cell numbers (Supplementary Figure 4) where they increased from the initial 2.4 × 10^3^ to 19–29 × 10^3^ cells ml^−1^ in treatment-specific fashions (Supplementary Figure 4). Representative images of Cryptophyta, Katablepharydophyta and CRY1 lineage with ingested bacterial prey are presented in Fig. 6.

### Grazing impact of HNF community at time zero

The cell-specific bacterivory rate, averaged for all HNF in the reservoir, was 13.7 bacteria HNF^−1^h^−1^ at the experimental start (*T*_0_), corresponding to a removal of 54.2% of the bacterial-standing stock per day (Table 3). Based on the number of ingested bacteria in food vacuoles targeted by a general bacterial probe, the cell-specific uptake rate of colorless Cryptophyta targeted by probe CryptB was even slightly higher (14.8 bacteria HNF^−1^ h^−1^) than the average for the total HNF (13.7 bacteria HNF^−1^ h^−1^). Notably, due to their high proportion of total HNF, Cryptophyta were also the most important bacterivores in the reservoir plankton at *T*_0_, accounting for ~70% of total HNF bacterivory (Table 3). In contrast, flagellates affiliated to the CRY1 lineage had markedly lower uptake rates and abundances, and thus also contributed correspondingly less to the bulk HNF bacterivory.

## Discussion

Our study demonstrates that bacterial prey characteristics differently affect growth and community dynamics of natural freshwater bacterivorous flagellates. This was evident from both prey-specific HNF growth parameters and taxonomic shifts in flagellate communities (Figs. 1, 2, 3, 4). We are aware of the fact that the concentrations of offered bacterial prey were far higher than the typical in situ concentrations, which could accelerate HNF growth and thus also influence growth parameters of HNF. However, even this increase in prey availability induced comparable DTs of HNF as those detected in dialysis bag experiments conducted directly in situ in the reservoir plankton with natural HNF and bacterial concentrations [43, 54].

### Growth parameters of HNF related to bacterial food quality

Very short lag phases of HNF (<3 h) in all treatments imply that the indigenous HNF community from the reservoir responded almost immediately to the offered bacterial prey, which is not always the case (compare the data in refs. [41] and [54]). Further, size ratios between offered cell size of bacteria and the size of natural HNF grazers indicated that all prey items were well within the edible size range for the grazers ([77]; [12, 78]; [79]).

A combination of short lag phase and rapid DT has been suggested as indication of high food quality of certain bacterial prey for natural HNF communities [20]. In our experiment, such a combination was well exemplified by the significantly shorter DTs and short lag phases detected in Rim11 and Rim47 treatments (Fig. 2a,c). Moreover, the relative growth rates of flagellate feeding on these medium-sized *Limnohabitans* strains were also significantly higher compared to the three other bacterial strains (Fig. 2d). On the other hand, large variability in triplicates for GGE estimates (28–39%) did not yield clear significant differences among treatments. However, a significant inverse relationship between the length of lag phase and GGE of flagellate communities was evident in a large data set with more diverse HNF communities and bacterial prey-specific characteristics [41, 54].

### Mismatch between 18 S rRNA amplicon data and cell abundances quantified by CARD-FISH

HTS allowed deeper and more detailed insights in the diversity of aquatic eukaryotes [21, 23, 80, 81] which, however, may not necessarily reflect an accurate estimation of the abundance of specific groups [82, 83]. Our study confirmed these concerns since relative abundances of reads belonging to Cryptophyta and Katablepharidophyta did not match at all with the relative abundances of cells detected by CARD-FISH in the same samples (Figs. 3, 4, 5). Such discrepancies could be explained by PCR biases of molecular approaches targeting single genes resulting in overestimations or underestimations of some groups [26, 27, 32, 84]. In this study, we also used a high number of PCR cycles (i.e., 35), which is at the upper range of recommended values, however, yet being within the normal range. This methodical aspect might perhaps partially contribute to the high discrepancy between amplicon and CARD-FISH results. Further, some hypervariable regions of 18 S rRNA, like V4 or V9, have been shown to be better for the estimation of certain groups [25].

In our study, proportions of reads affiliated to Katablepharidophyta were drastically overestimated compared to CARD-FISH counts, which could be related to high numbers of rRNA operon copies in this group [85]. Copy numbers of 18 S rRNA genes can vary among different protistan taxa depending on the cell and genome size [86]. Phylogenetic position of Katablepharidophyta is still under debate and for long time they have been considered as a part of either Cryptophyta or Alveolata [87], with the latter group being known to possess very high copy numbers of 18 S rRNA genes [82]. However, few phylogenetic analyses confirmed their position as a sister group to Cryptophyta [88, 89]. We can exclude a taxonomic mis-assignment of short reads from amplicon sequencing, as two of the most abundant OTUs were clearly affiliated to Katablepharidophyta (Supplementary Fig. 3) and the sequence of our newly designed CARD-FISH probe targets the V9 region that is present in all reads (Table 2).

On the other hand, numbers of Cryptophyta were drastically underestimated with HTS, which might be due to primer bias as some publically available sequences for Cryptophyta have mismatches with primers that we used in this study (for more details see Supplementary Table 1).

Although we found large mismatch between HTS and CARD-FISH results, these two methods combined together provide a powerful tool to detect diversity and abundance of certain groups. Amplicon sequencing can be especially useful for identifying taxa present in a large set of samples and facilitates designing of new CARD-FISH probes. The application of group specific primers [32], or the carefull design of new primers can decrease certain biases in amplicon sequencing. CARD-FISH on the other hand, is a very valuable method for a more accurate estimation of abundance of specific lineages since it is possible to visualize and thus directly quantify target organisms. However, CARD-FISH has its downsides and limitations. It is very laborious and limited in the number of taxon specific probes that could be applied at the same time [69]. Further, it is not possible to accuratelly estimate the abundances of rare taxa with CARD-FISH, while HTS can still detect them.

### Cryptophyta—unexpected major bacterivores

Our study documents a strong impact of prey characteristics on resulting HNF community dynamics, with severe shift in HNF community composition towards Cryptophyta (Fig. 3). Furthermore, flagellates belonging to Cryptophya were the most abundant bacterivores in summer plankton of the Řimov reservoir, which was confirmed by high cell-specific grazing rates making them responsible for 70% of total HNF bacterivory (Table 3). Additionally, they undoubtedly grew and feed on all the tested bacterial strains in our experiments as documented in the double hybridization of grazers and prey (Fig. 6).

In the past decade, numerous studies suggested that the most important bacterivores in freshwaters belong to small colorless chrysomonad flagellates, so called “*Spumella*-like” flagellates ([20, 21]; 90; [8, 91]). The term “*Spumella*-like” is mostly used when addressing morphology of these flagellates as recently it has been shown that they are in fact polyphyletic, belonging to different groups of the class Chrysophyta [23, 80]. Chrysophyta reads accounted for >2% of the flagellates collected in situ (*t*_0_ h) and increased to 3–9% after 40 h of experiment. A significant increase in two treatments, Rim47 and Rim11, indicated efficient growth of chrysomonad flagellates on these two strains (Fig. 3). Since we did not use a specific CARD-FISH probe for this group we cannot confirm agreement with the abundance estimates based on amplicon reads. Interestingly, those results partly contrast to a similar study conducted by Šimek et al. [20] scheduled to the spring bloom phase (late April) in the Římov reservoir, where the majority of reads were associated with different lineages of “*Spumella*-like” flagellates. Our experiment was conducted in late summer, suggesting that seasonal aspects strongly modulate the community of eukaryotes and that different flagellate taxa are likely to be major bacterivores in different seasons [49, 54]. The most abundant bacterivores in our experiments, according to CARD-FISH results, were affiliated to Cryptophyta (Fig. 4; Supplementary Figure 4). The recently discovered CRY1 clade of Cryptophyta [92] appears to be an important bacterivore in our study, growing on all tested strains but with a profound increase only on the bacterial strain Rim11. Cells of flagellates belonging to this clade were relatively small (~3–4 µm length), spherical and with a de-central nucleus (Fig. 6). All observed cells were purely heterotrophic with no chloroplasts (as previously suggested by Piwosz et al. [35]) but having ingested bacteria in their food vacuoles.

Since phylogenetic resolution of amplicon sequencing is low and most Cryptophyta were considered to be either phototrophic or mixotrophic [14, 30], the CRY1 clade and other heterotrophic cryptophytes (Table 3) were so far largely overlooked as potentially bacterivorous. However, abundances of other Cryptophyta cells not belonging to the CRY1 lineage, yet being targeted by the general Cryptophyta probe CryptB [75] were unexpectedly high (Figs. 4, 5, 6; Supplementary Figure 4). Relative abundances up to 70% of all eukaryotic cells, with, moreover, high cell-specific uptake rates (Table 3), suggest the existence of additional heterotrophic bacterivorous clades among this phylum. This is in agreement with a recent study by Debroas et al. [15], which reported several unknown lineages of Cryptophyta. Cells targeted by the general Cryptophyta probe had diverse morphologies and food vacuoles containing numerous bacterial prey (Fig. 6c-d). Notably, prior to the experiment (*T*_0_), Cryptophyta had higher uptake rates compared to total HNF and to the CRY1 lineage (Table 3). Thus, they were the most abundant bacterivores already in situ, which was not reported before. Chloroplasts were never observed in these flagellates although we cannot confirm their obligate heterotrophy since the strong signal of the probe might slightly interfere with the chlorophyll *a* excitation. However, the majority of HNFs in our experiment were aplastidic, as almost no chloroplast bearing flagellates (<2%) were observed in DAPI-stained preparation.

On the other hand, Katablepharydophyta have not been observed with ingested bacteria in our experiments (Fig. 6a) and their numbers increased significantly only towards the end of the experiment (Fig. 4; Supplementary Figure 4). Thus we cannot exclude the possibility that they fed on smaller bacterivorous HNF. This would correspond to the fact that some species of Katablepharidophyta are known to prey on a large prey such different types of algae [93] and a peculiar way of feeding by forming swarms was observed in some cultures [89, 93].

### Double hybridization as a powerful method for studying bacterivory

Our study demonstrated that the combination of high-throughput amplicon sequencing with the design of specific CARD-FISH probes can serve as a powerful tool for estimating diversity and quantifying abundance of prevailing distinct eukaryotic groups. Moreover, we applied a new method (see also ref. [55]) for examining bacterivory by combining two probes at different trophic levels, targeting protistan grazers as well as prey bacteria in their food vacuoles (Fig. 6). This combination gives new insights into predator–prey interactions as it displays a unique picture in situ, by demonstrating directly which bacteria are preferentially consumed and which groups of flagellates are their grazers in aquatic ecosystems at a given time.

## Conclusions

The design and application of novel eukaryotic probes for CARD-FISH has been fundamental to our study, as we could quantify and elucidate the trophic mode of the CRY1 clade of Cryptophyta, discovered by Shalchian-Tabrizi et al. [92]. This group appeared to be an important bacterivore in summer plankton communities, feeding and growing well on several betaproteobacterial strains, but most profoundly on one strain of *Limnohabitans* in our experiment (Fig. 4). To our best knowledge the CRY1 group has so far not been observed with ingested bacteria nor has their bacterivory ever been quantified. Thus, our study clearly evidenced the key role of bacterial food as carbon source fueling growth of these small protists as suggested earlier [35]. Further, flagellates targeted by a general Cryptophyta probe were the most abundant bacterivores not only in all our prey-amended treatments but also in situ in Římov reservoir (Table 3). For the first time we could visualize this finding via a double hybridization method that allowed for a simultaneous phylogenetic identification of both grazers and prey without additional sample manipulation (Fig. 6). Moreover, we could also demonstrate that a quantification based solely on numbers of reads by HTS is insufficient to accurately estimate abundances of certain groups, as exemplified for Katablepharidophyta and Cryptophyta. Last but not least, our study clearly demonstrated species-specific effects of the prey quality on the resulting community composition of HNF.

## Electronic supplementary material

Supplement material

Data set 1
