# Supplementary material for: Cryptophyta as major bacterivores in freshwater summer plankton
Source: ISME J. 2018 Feb 20;12(7):1668–81. doi: 10.1038/s41396-018-0057-5 (PMC6018765; doi:10.1038/s41396-018-0057-5)
Supplement: Supplementary file 1 — Supplement material [file 41396_2018_57_MOESM1_ESM.doc]

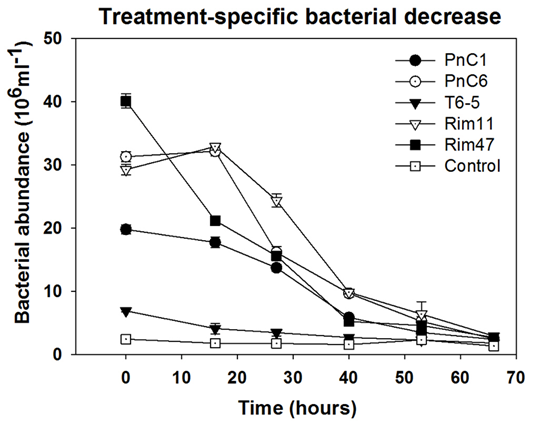


Supplementary Figure 1. Bacterial numbers in experimental treatments amended with bacterial strains (PnC1, PnC6, T6-5, Rim11 and Rim47) compared to the control where no bacterial strains were added at different times of the experiment. Values are means of triplicates. Error bars depict standard deviations.


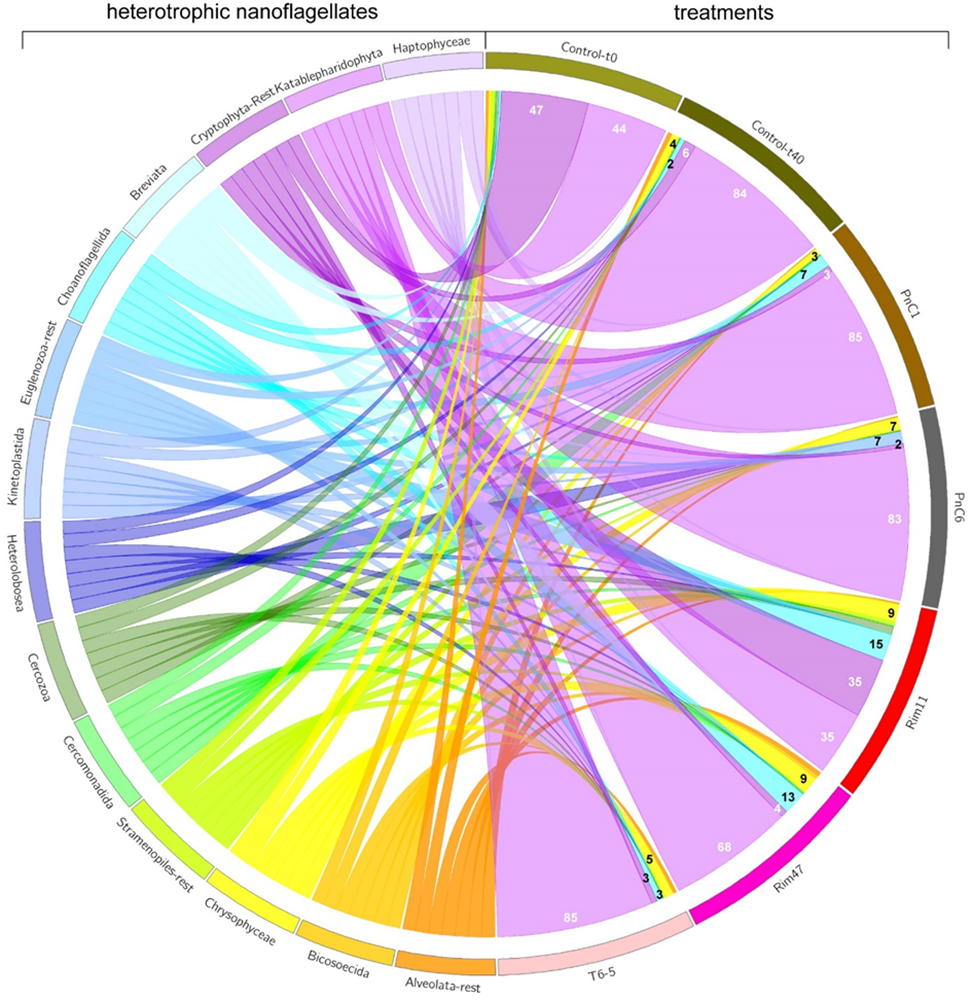


Supplementary Figure 2. Percentages of reads affiliated with particular taxonomical groups of protists in different treatments and time points. The left half of the circus plot shows the occurring flagellate groups; the right half shows the treatments. Control t0 represents the starting community from the reservoir. Control t_40_ represents the control treatment after 40 hours of experiment, PnC1, PnC6, T6-5, Rim47, and Rim11 show the protistan community in the bacterial prey-amended treatments after 40 hours of experiment. Values are means of triplicates expressed as percentages.


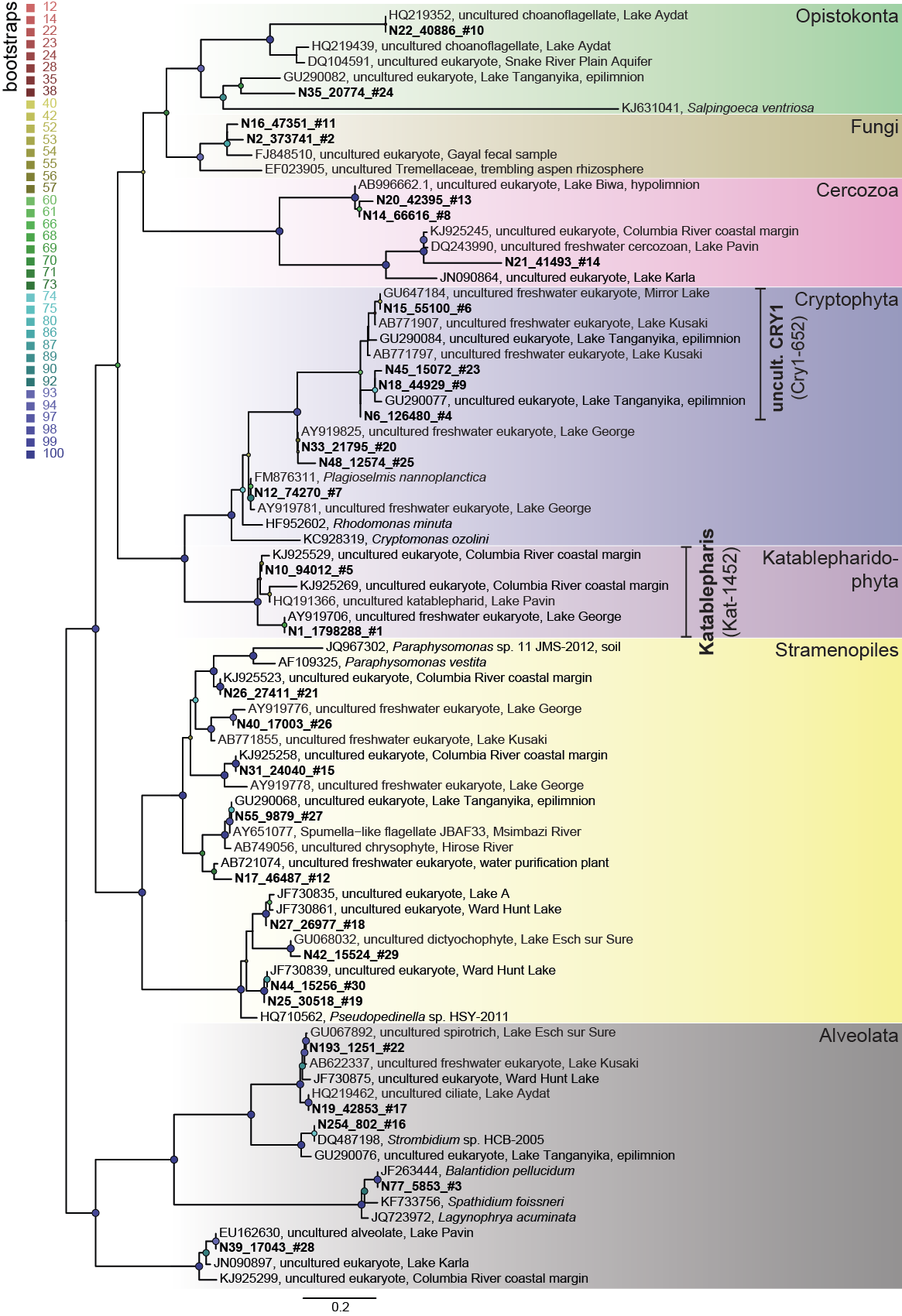


Supplementary Figure 3. Bootstrapped maximum likelihood tree of eukaryotic 18S rRNA genes including representative sequences of the 30 most abundant OTUs from the amplicon dataset (marked in bold; OTU rank is indicated by #). Sequences targeted by the newly designed probes Cry1-652 and Kat-1452 are shown in brackets. Bootstrap values are indicated by differentially colored circles on nodes, the scale bar at the bottom applies to 20% sequence divergence.


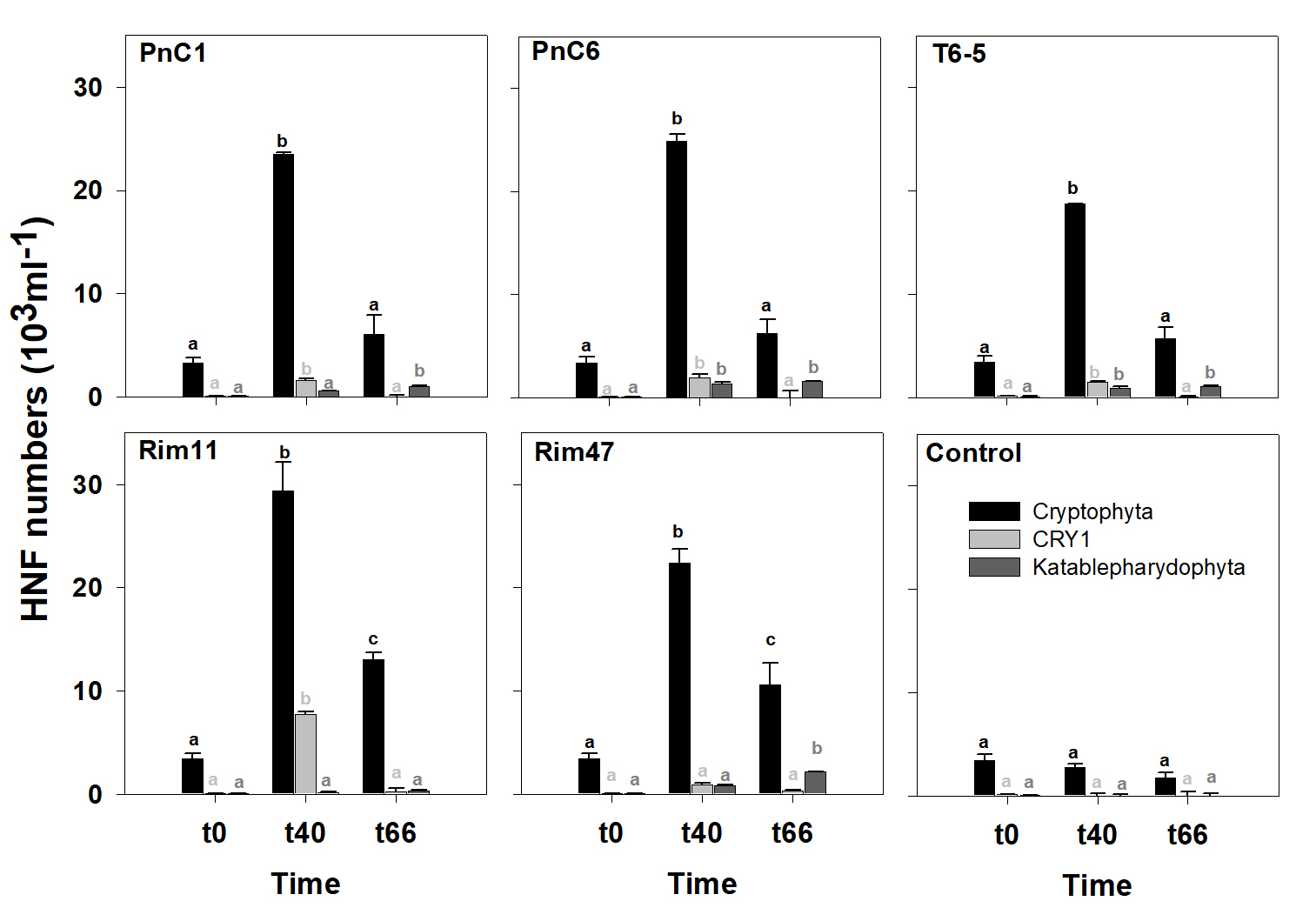


Supplementary Figure 4. Absolute abundances of HNF cells hybridized with probes targeting all Cryptophyta, lineage CRY1, and all Katablepharidophyta at three different time points: t_0_, beginning of experiment, representing the starting community from the reservoir; t_40_ and t_60_ represent percentage after 40 and 60 hours of experiment. Different letters above the columns indicate significant differences between different times of the experiment within one treatment targeted with one probe (post hoc Tukey test). Values are means of triplicates.
